# Circular RNA as Therapeutic Targets in Atherosclerosis: Are We Running in Circles?

**DOI:** 10.3390/jcm12134446

**Published:** 2023-07-02

**Authors:** Jeffrey Triska, Christo Mathew, Yang Zhao, Yuqing E. Chen, Yochai Birnbaum

**Affiliations:** 1 Department of Medicine, Baylor College of Medicine, Houston, TX 77030, USA; 2 Cardiovascular Center, Department of Internal Medicine, University of Michigan Medical Center, Ann Arbor, MI 48109, USA; 3 Section of Cardiology, Department of Medicine, Baylor College of Medicine, Houston, TX 77030, USA

**Keywords:** circular RNA, micro RNA, atherosclerosis, cardiovascular disease, potential therapeutic treatments

## Abstract

Much attention has been paid lately to harnessing the diagnostic and therapeutic potential of non-coding circular ribonucleic acids (circRNAs) and micro-RNAs (miRNAs) for the prevention and treatment of cardiovascular diseases. The genetic environment that contributes to atherosclerosis pathophysiology is immensely complex. Any potential therapeutic application of circRNAs must be assessed for risks, benefits, and off-target effects in both the short and long term. A search of the online PubMed database for publications related to circRNA and atherosclerosis from 2016 to 2022 was conducted. These studies were reviewed for their design, including methods for developing atherosclerosis and the effects of the corresponding atherosclerotic environment on circRNA expression. Investigated mechanisms were recorded, including associated miRNA, genes, and ultimate effects on cell mechanics, and inflammatory markers. The most investigated circRNAs were then further analyzed for redundant, disparate, and/or contradictory findings. Many disparate, opposing, and contradictory effects were observed across experiments. These include levels of the expression of a particular circRNA in atherosclerotic environments, attempted ascertainment of the in toto effects of circRNA or miRNA silencing on atherosclerosis progression, and off-target, cell-specific, and disease-specific effects. The high potential for detrimental and unpredictable off-target effects downstream of circRNA manipulation will likely render the practice of therapeutic targeting of circRNA or miRNA molecules not only complicated but perilous.

## 1. Introduction

Much attention has been paid lately to harnessing the diagnostic and therapeutic potential of non-coding circular ribonucleic acids (circRNAs) and micro-RNAs (miRNAs) for the prevention and treatment of cardiovascular diseases. The advent of next-generation sequencing and the development of bioinformatics databases have facilitated the rapid expansion of circRNA research [1]. One recent review by Wang et al. [2] suggests that, although further clinical trials and basic scientific research are needed, targeting cardiovascular disease pathways via circRNA-mediated mechanisms may prove to be an efficacious strategy for preventing and diagnosing cardiovascular diseases [2]. A large body of ongoing research involves the roles of circRNA molecules in atherosclerosis, the underlying condition contributing to most cardiovascular diseases and the leading cause of death in the world.

The desire to attenuate the progression of atherosclerosis at the genetic level is appealing. Gene regulation likely plays a large role in the pathogenesis of atherosclerosis [3]. By understanding and manipulating the genetic pathways involved in atherosclerosis, we may be able to develop novel therapeutic targets, potentially including drugs for primary prevention. This would be a momentous development; given the recent demotion of chronic low-dose aspirin use, there are limited treatment options beyond those of lifestyle and risk factor modification for the primary prevention of cardiovascular disease. Still, 48% of adults older than 20 years of age have cardiovascular disease and would benefit from additional therapeutic approaches [4].

However, the genetic environment that contributes to atherosclerosis pathophysiology is immensely complex. A review by Siebert et al. [5], which summarized the role of non-coding RNAs, including miRNAs, in ischemic myocardial reperfusion injury, warned of a vast interplay between all non-coding RNAs, including circRNAs, that can have far-reaching effects throughout the entire body. Therefore, any potential therapeutic application of circRNAs must be assessed for risks, benefits, and off-target effects in both the short and long term [5]. Through a comprehensive review and analysis of the literature, this paper aims to evaluate the potential of circRNAs as therapeutic targets in atherosclerosis.

## 2. Methods

A search of the online PubMed database for publications from 2016 to 2022 was conducted using the following keywords: circular RNA, atherosclerosis, coronary artery disease, oxidized low-density lipoprotein, vascular smooth muscle cells, and endothelial cells. A total of 140 original research articles were included, and their findings are summarized in Table S1 [6,7,8,9,10,11,12,13,14,15,16,17,18,19,20,21,22,23,24,25,26,27,28,29,30,31,32,33,34,35,36,37,38,39,40,41,42,43,44,45,46,47,48,49,50,51,52,53,54,55,56,57,58,59,60,61,62,63,64,65,66,67,68,69,70,71,72,73,74,75,76,77,78,79,80,81,82,83,84,85,86,87,88,89,90,91,92,93,94,95,96,97,98,99,100,101,102,103,104,105,106,107,108,109,110,111,112,113,114,115,116,117,118,119,120,121,122,123,124,125,126,127,128,129,130,131,132,133,134,135,136,137,138,139,140,141,142,143,144,145].

These studies were reviewed for their design, including methods for developing atherosclerosis and the effects of the corresponding atherosclerotic environment on circRNA expression. Investigated mechanisms were recorded, including associated miRNA, genes, and ultimate effects on cell mechanics, inflammatory markers, etc. Study findings were assessed for whether they were likely protective or promoted atherosclerosis development. This determination was made based on the association of known mechanisms associated with atherosclerosis pathogenesis. Accordingly, enhanced proliferation, migration, apoptosis, inflammation, oxidative stress, and pathogenic particle uptake were considered harmful, while the opposite was considered protective. Studies with both presumed protective and harmful effects were deemed to have equivocal findings. The most investigated circRNAs were then further analyzed for redundant, disparate, and/or contradictory findings.

## 3. Results

This review identified 140 studies conducted between 2016 and 2022 (Table S1)**.** The majority employed in vitro models of human vascular smooth muscle cells (VSMCs) or endothelial cells (ECs). Atherosclerosis was simulated by stimulating cells with known pathogenic triggers, with oxidized low-density lipoprotein (ox-LDL) being the most common. Other triggers include platelet-derived growth factor-BB (PDGF-BB), high glucose, or high-fat diets in in vivo models. The effects of these pathogenic states on circRNA, miRNA, and their associated gene expression were then analyzed, as were their effects on cell proliferation, migration, apoptosis, inflammation, and oxidative stress. The interaction of these genetic molecules and their effects of expression on cell behavior and inflammation were elucidated via various assays, especially immunohistochemical staining techniques, after silencing the molecules of the presumed pathway. Only 19 (13.6%) studies performed an ancillary in vivo mouse/rat model. More commonly, the particular circRNA or miRNA level under investigation was measured in the serum of human subjects or mice with atherosclerosis to corroborate the experimental findings in vitro.

**Figure 1:**
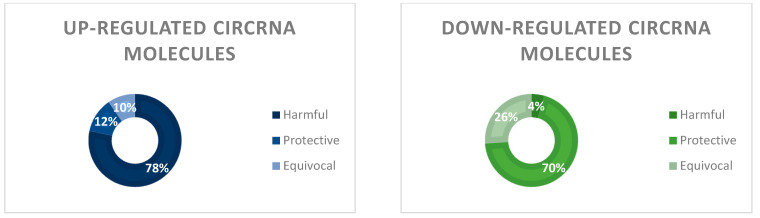
The association between the overall effects of circRNA expression on the process of atherosclerosis. Up-regulation of circRNAs in atherosclerosis was found to be most associated with harmful (78%) effects, while downregulation was most associated with protective mechanisms (70%). Equivocal effects were demonstrated in 10% and 26% of studies in which circRNAs were observed to be up-regulated and downregulated, respectively.

Of the 140 studies reviewed, 95 unique circRNA molecules were identified. The majority (76.8%) were up-regulated in in vitro atherosclerotic environments and/or the serum of patients with atherosclerosis. Of these, 79% correlated with mechanisms known to have pro-atherosclerotic effects in vivo, while 12.3% were associated with protective mechanisms. Of the 25.3% of cirRNAs found to be downregulated, overexpression of these molecules was more commonly associated with mitigation (69.6%) than propagation (4.3%) of atherosclerosis-associated mechanisms. One circRNA molecule, circHIPK3, was shown to be both up-regulated and downregulated across different studies [37,61,82,143]. Of note, at least 9.6% of circRNAs that were demonstrated to have increased expression had equivocal outcomes, i.e., they were unable to discern if the overall effects observed were pathogenic or protective. This percentage was even higher (26.1%) when analyzing only the molecules that were downregulated by atherosclerotic stimuli in vitro (Figure 1).

The overwhelming majority of studies showed that circRNA molecules exerted their effects via sponging a cognate miRNA (Table S1). In turn, this led to increased expression of a particular gene and subsequent effects on cell mechanics and inflammatory pathways. Only 10 of the 140 studies demonstrated that circRNAs either executed their effects through a mechanism independent of miRNA sponging or failed to identify an associated miRNA [6,9,22,25,29,32,51,52,66,106]. Additionally, of the 133 miRNA molecules identified, 102 were unique, with the corollary being that 23.3% of miRNA molecules were found to interact downstream of multiple circRNAs. To highlight the redundancies and issues with replicability of these studies, the experimental findings of the most investigated molecules are discussed below, with attention paid to opposing or equivocal findings and mechanistic overlap.

### 3.1. CircANRIL

One of the first circRNAs discovered to play a role in atherosclerosis was circANRIL (Antisense non-coding RNA in the INK4 locus). It is located on chromosome 9p21, variants of which are known genetic risk factors for developing cardiovascular disease [146]. In 2010, Burd et al. [147] demonstrated that homozygous individuals for the atherosclerotic risk allele showed decreased expression of circANRIL and the coding INK4/ARF transcripts [147]. CircANRIL was later found to impair ribosome biogenesis, leading to activation of p53, which then resulted in decreased proliferation and increased apoptosis by directly binding to PES1 (pescadillo ribosomal biogenesis factor 1), an essential 60S-preribosomal assembly factor in VSMCs, ECs, and adventitial fibroblasts [6]. This represents one of the rare instances discovered in which circRNAs modulate atherosclerotic events via transcriptional regulation rather than indirectly through miRNA sponging. However, whether inhibition of cellular proliferation or apoptosis ultimately has positive or negative effects on atherosclerosis development depends on various factors that are difficult to determine with certainty [6].

Other studies attempted to corroborate the effects of circANRIL expression in an in vivo mouse model of atherosclerosis. Song et al. [9] showed that circANRIL overexpression was associated with the formation of atherosclerotic plaques and thombi in rats that were fed a high-fat diet and injected with a large dose of vitamin D3 to promote arterial calcification [9]. The study further supported the findings of increased rates of apoptosis demonstrated by Holdt et al. [6], but also showed higher levels of total cholesterol, triglycerides, LDL, and several pro-atherosclerotic and inflammatory markers, including interleukin (IL)-1, IL-6, matrix metallopeptidase-9 (MMP-9), c-reactive protein (CRP), BCL2 associated X (Bax), and caspase-3 [9]. Another investigation demonstrated that inhibition of circANRIL expression in a similar in vivo rat model reduced markers of vascular endothelial injury, oxidative stress, and inflammation [148]. These early studies suggested that in vivo models analyzing plaque development and inflammatory markers may produce reliable results to establish a causal link between circRNA expression and atherosclerosis development.

### 3.2. Circ_USP36/Circ_0003204

This review identified 11 different studies evaluating the role of circ_USP36 (ubiquitin specific peptidase 36)/circ_0003204 in the pathogenesis of atherosclerosis, establishing it as the most investigated circRNA molecule. All experiments were conducted in in vitro models of human VSMCs and ECs [29,42,55,72,74,75,78,81,92,98,139]. Liu et al. [29] showed that hsa_circ_0003204 was aberrantly overexpressed in ox-LDL-induced human umbilical vein ECs (HUVECs), while knockdown of this molecule promoted proliferation, migration, and invasion but reduced apoptosis [29]. Reduced expression of circ_0003204 also significantly correlated with lower E-cadherin but increased activity of N-cadherin and vimentin in oxLDL-induced HUVECs, findings that are associated with reduced cell mobility and plaque stability, respectively [149,150]. Thus, the knockdown of circ_0003204 was associated with both increased (harmful) and decreased (protective) cellular proliferation in this study. No associated miRNA was identified in this particular study.

Several other in vitro experiments demonstrated suppressed cell viability and promotion of apoptosis, inflammation, oxidative stress, and cell migration and invasion associated with increased expression of circ_USP36 and subsequent miRNA sponging in response to ox-LDL-stimulated ECs. Specifically, these effects were attributed to circ_USP36/circ_0003204 inhibition of miR-20a-5p, miR-98-5p, and miR-188-3p leading to increased ROCK2, vascular cell adhesion protein-1 (VCAM-1), and TRP6 gene expression, respectively [72,74,75,81,92]. On the other hand, Huang et al. [55] observed that, through increased expression of WNT4 from sponging of miR-637, circ_USP36 overexpression was associated with suppressed proliferation and migration of human aortic ECs treated with ox-LDL in vitro [55]. While largely agreeing with the effects that circ_USP36/circ_0003204 promotes inflammation and oxidative stress, a recent study also found decreased tube formation in HUVECs stimulated with ox-LDL through sponging of miR-491-5p and increased expression of intercellular adhesion molecule-1 (ICAM-1) [139]. Taken together, these studies present contradictory results regarding the effects of circ_USP36/circ_0003204 on VSMC and EC proliferation, migration, and invasion, suggesting that their regulation is complex and clinical significance challenging to capture.

**Figure 2:**
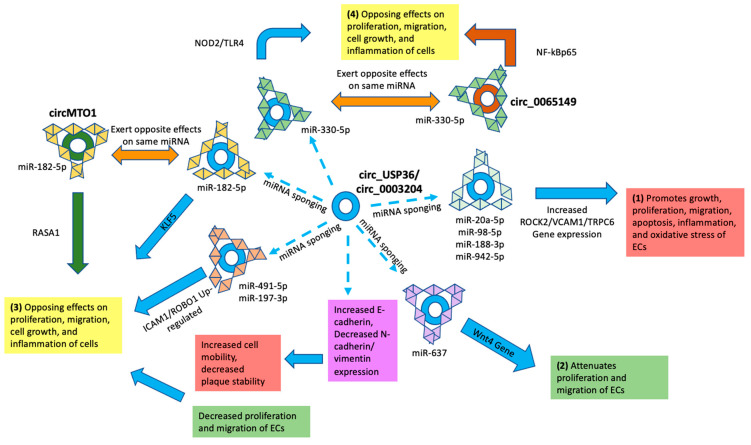
The role of circ_0003204/USP36 in the pathogenesis of atherosclerosis. Sponging of miRNAs by up-regulated circ_0003204/USP36 has been shown to lead to (1) promotion of growth, proliferation, migration, apoptosis, inflammation, and oxidative stress of ECs [72,74,75], but also (2) attenuation of proliferation and migration of ECs, known to be protective from further intimal hyperplasia [55]. Furthermore, some miRNAs are inhibited by multiple circRNAs, the effects of which have (3) and (4) contradictory outcomes on cell growth, proliferation, migration, and inflammation [29,42,78,81,92,98,139]. These opposing effects make it difficult to predict the overall effects of targeting a specific circRNA or miRNA for therapeutic purposes.

Another study showed that the expression of circ_USP36 was also increased in ox-LDL-treated human umbilical vein VSMCs via sponging of miR-182-5p [42]. This led to increased activity of the KLF5 gene, which induced VSMC proliferation and metastasis. Circ_USP36 knockdown inhibited this proliferation and metastasis by up-regulating miR-182-5p [42]. However, lower levels of circMTO1 in the serum of humans with atherosclerosis coincided with augmentation of miR-182-5p, increased proliferation, and reduced apoptosis in an in vitro analysis of ox-LDL-stimulated VSMCs. Overexpression of circMTO1 led to less inhibition of miR-182-5p and subsequently greater activation of the RASA1 gene, reduced proliferation, and increased apoptosis of VSMCs [57]. Similarly, while lower levels of circ_0065149 were observed in a model of ox-LDL human umbilical vein ECs in vitro, overexpression was associated with miR-330-5p sponging and associated effects of increased cell viability, proliferation, and migration, but reduced apoptosis and inflammation [62]. These outcomes are opposed to those of increased inflammation with miR-330-5p sponging observed by Su et al. [78]. These studies demonstrate that different circRNA molecules can exhibit both protective and detrimental effects on the development of atherosclerosis via sponging of the same miRNA. This suggests that targeting a specific circRNA for therapeutic purposes could possibly result in unintended pathogenic consequences in opposition to the objective of such manipulation (Figure 2).

### 3.3. CircCHFR

Six in vitro experiments examined circCHFR [21,43,85,112,117,128]. All studies showed consistent results of upregulation of circCHFR in atherosclerotic environments simulated by treating cells with ox-LDL or PDGF-BB. Subsequent miRNA sponging and overexpression of various genes were further associated with pro-atherosclerotic mechanisms. In one model, sponging of miR-370 led to increased FOXO1/Cyclin D1 expression, facilitating VSMC proliferation and migration [21]. Another in vitro study linked these findings with increased markers of inflammation via miR-214-3p inhibition and increased Wnt4 expression [43]. Increased circCHFR activity was also associated with the augmentation of apoptosis and proinflammatory cytokine secretion. Reciprocally, silencing of circCHFR increased cell survival and reduced apoptosis in ECs [112]. When analyzed at the level of circRNA expression alone, these studies appear to show that up-regulation of circCHFR in models of atherosclerosis consistently and reliably leads to pathogenic progression.

**Figure 3:**
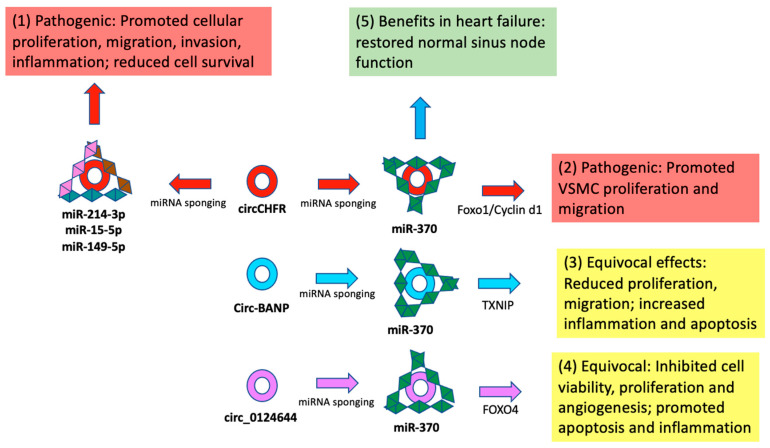
The role of circCHFR in the pathogenesis of atherosclerosis. Sponging of several miRNAs by circCHFR has been shown to lead to (1) increased cellular proliferation, migration, invasion, inflammation, and reduced cell cycle survival—all mechanisms known to contribute to atherosclerosis development [43,85,128]. However, circCHFR has also been demonstrated to (2) reduce the expression of miR-370 [21], the inhibition of which has also been shown to have opposing effects compared to circCHFR via sponging by (3) circ-BANP and (4) circ_0124644 [45,121]. (5) Disinhibition of mir-370 also likely adversely affects sinus node function in patients with heart failure [151].

However, miR-370 has been found to be regulated by other circRNA molecules with opposing downstream consequences. For example, sponging of miR-370 by circ-BANP has been associated with reduced proliferation and migration of HUVECs, the opposite of that found through the interaction of cricCHFR and miR-370 [21,45]. Overexpression of circ_0124644 leading to inhibition of miR-370 similarly had equivocal outcomes on atherosclerosis progression by inhibiting cell viability, proliferation, and angiogenesis but promoting apoptosis and inflammation [121]. Silencing of miR-370 has also been associated with sinus node function recovery in patients with heart failure [151]. These studies suggest contradictory effects via similar mechanisms of miR-370 inhibition (Figure 3).

Thus, therapeutic interventions aimed at reducing circCHFR expression may lead to conflicting results via downstream gene regulation and disparate effects on VSMC and EC proliferation and migration. Even if these effects ultimately reduce atherosclerosis progression, disinhibition of miR-370 may promote life-threatening arrhythmias in patients with heart failure, suggesting that cardiovascular pathologies other than atherosclerosis may also be negatively affected [151]. Furthermore, since these studies were not corroborated in in vivo models, it is hard to determine the ultimate effects of the mechanisms elucidated on the process of atherosclerosis. While most studies also observed that circCHFR was up-regulated in the serum of patients with atherosclerosis, this up-regulation may lead to the promotion of some genes that foster protective effects against atherosclerosis.

### 3.4. CircHIPK3

Four in vitro models studied the effects of circHIPK3 in atherosclerosis pathogenesis [37,61,82,143]. Two of these investigations showed higher levels of circHIPK3 in pro-atherosclerotic in vitro environments, while two demonstrated attenuated activity (Table S1). Wang et al. [82] showed that increased levels of circHIPK3 in mice aortic EC-secreted exosomes in response to high glucose levels correlated with more significant proliferation and inhibition of apoptosis of VSMCs and VCAM-1 expression and uptake of glucose-rich exosomes by VSMCs. This occurred via sponging of miR-106a-5p and amplified expression of FOXO1 and VCAM-1 [82]. Similar effects were seen in human aortic and umbilical artery VSMCs through a mechanism involving inhibition of miR-637, leading to increased expression of cyclin-dependent kinase 6 (CDK6) [61]. In opposition to these findings, sponging of miR-637 by circ_0002194 correlated with reduced angiogenesis and increased apoptosis rates of ox-LDL-treated vascular ECs [122].

**Figure 4:**
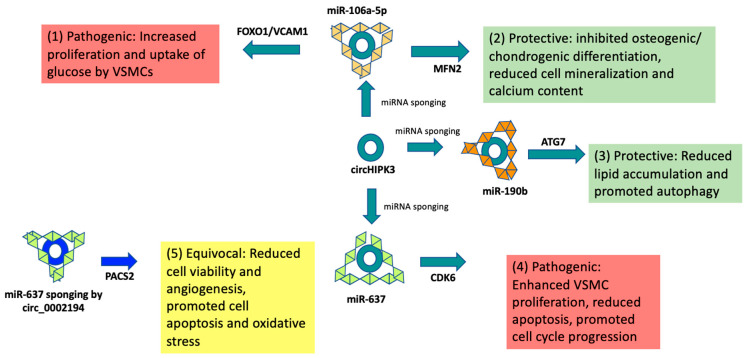
Studies investigating the effects of in vitro atherosclerosis environments on circHIPK3 demonstrate both (1) and (4) harmful effects of cellular proliferation, migration, and apoptosis [61,82], and (2) and (3) protective effects of inhibited osteogenic differentiation, mineralization, and calcium deposition, reduced lipid accumulation, and increased rates of autophagy [37,143]. Both (1) harmful and (2) protective effects were seen from sponging of miR-106a-5p [82]. Opposing effects on angiogenesis were also demonstrated via sponging of miR-637 by (4) circHIPK3 and [61] (5) circ_0002194 [122], the latter of which suggests equivocal effects on the pathogenesis of atherosclerosis due to the observed impaired angiogenesis but enhanced oxidative stress.

Zhang W-B et al. [143] found lower levels of circHIPK3 in the serum and tissues of patients with atherosclerosis. This was associated with osteogenic and chondrogenic differentiation and increased cell mineralization and calcium content in VSMCs in vitro. In fact, overexpression of circHIPK3 led to sponging of miR-106a-5p and subsequent activation of the MFN2 gene, which inhibited osteogenic and chondrogenic differentiation, ultimately leading to less calcium accumulation in VSMCs [143]. In this case, miR-106a-5p sponging had beneficial effects, which contradicts the findings that miR-106a-5p inhibition facilitated pathogenic proliferation and migration of VSMCs [82]. Another study showed that downregulation of circHIPK3 led to disinhibition of miiR-190b, decreased activity of the ATG7 signal pathway, and subsequently lower rates of autophagy and higher rates of lipid accumulation in both mice in vivo and ox-LDL-treated human umbilical vein ECs in vitro [37]. On the other hand, overexpression of circHIPK3 resulted in sponging of miR-190b and increased activity of the ATG7 pathway, which correlated with reduced lipid accumulation and promoted autophagy (Figure 4).

Analysis of the results of the circHIPK3 investigations illustrates three major points of contention: 1. In similar proxies for atherosclerotic environments, crcHIPK3 expression was found to be both increased and decreased. 2. Analysis downstream of circHIPK3 expression, i.e., of miR-637, showed opposing effects when it was inhibited by other circRNA molecules, i.e., circ_0002194. 3. The ultimate effects of overexpression of circHIPK3 were found to be both pathogenic (increased proliferation, apoptosis, and glucose uptake) and protective (reduced angiogenesis, apoptosis, osteogenic differentiation, and lipid accumulation) in regard to atherosclerosis development and progression. It is possible disparate effects were seen due to different tissue types and methods of atherosclerosis stimulation in vitro. However, similar results would have been expected regardless of the method used to simulate atherosclerosis. Furthermore, the possibility that intervening in one tissue type to halt atherosclerosis progression could promote atherosclerosis in a different tissue type is alarming. Alternatively, these findings could point to issues with the general replicability of these studies in vitro. Regardless, they underscore the complexity of the genetic milieu of atherosclerosis and the likely unintended negative consequences of circRNA manipulation.

## 4. Discussion

Several issues have been raised with using circRNAs as potential therapeutic targets to modify disease processes. Some systemic problems include the toxicity of nanoparticles, mis-spliced byproducts, and synthetic circRNA immunogenicity [152]. Highlighted by this review—with respect to in vitro models evaluating atherosclerosis—are also questions of study design, interpretation of overall results, contradictory effects caused by off-target RNA silencing, and cell-specific and disease-specific effects. No two studies that used a supplemental in vivo model studied the same circRNA. Thus, we cannot comment on the redundancy or reproducibility of the effects of a particular circRNA within in vivo models.

### 4.1. Study Design and Issues with Interpretation

As previously discussed, the majority of the circRNA molecules that were studied were upregulated in the serum of subjects with atherosclerosis and in in vitro models of atherosclerosis induced via established pathogenic triggers. This is likely because circRNA molecules with increased levels are easier to identify than ones with decreased expression, which represents a kind of ascertainment bias in identifying potential circRNA targets for investigation. The majority of experiments, which found increased expression of circRNA molecules in states of atherosclerosis, also found an association with mechanisms known to promote atherosclerosis in vivo—most commonly proliferation and apoptosis of ECs or VSCMS—while silencing the circRNA under investigation and promoting its cognate miRNA resulted in opposite effects. However, whether angiogenesis and apoptosis in atherosclerosis are beneficial or harmful depends on their effects on intimal hyperplasia, plaque stability, plaque content, phenotypic switching, and the stage of atherosclerosis [153,154]. Thus, it is difficult to determine the clinical significance of atherosclerotic mechanisms in vitro.

Even when conceding the benefit of the doubt that a particular mechanism known to promote atherosclerosis in vivo, e.g., proliferation and migration of EC and VSMCs, has similar effects in vitro, a significant percentage of studies yielded equivocal results. Often, seemingly opposing effects were observed in response to upregulation or downregulation of a particular circRNA in vitro. For example, Chen et al. [45] demonstrated that while circ-BANP was associated with apoptosis and inflammation and promoted cell viability, it also correlated with increased migration, invasion, and tube formation of ECs [45]. Antagonistic effects on proliferation, migration, and promotion of calcification of VSMCs by circHIPK3 sponging of miR-106a-5p were also observed (Figure 4). Whether these mechanisms, which have seemingly oppositive effects on atherosclerosis development, lead to the progression or attenuation of atherosclerosis in toto, it is difficult to ascertain via in vitro analyses alone. EC dysfunction present in the early stages of atherosclerosis is associated with chronic inflammatory changes in the arteries [155]. Alternatively, the results could have been inaccurate, pointing to potential issues with the general replicability of the results of these study designs.

Ancillary in vivo studies often investigate different pathogenic processes or stages of atherosclerosis and therefore do not effectively corroborate the in vitro findings. For example, one study that evaluated the effects of circGSE1 expression on EC senescence also looked at the effects of angiogenesis on limb ischemia in mice via femoral artery ligation [125]. Few studies have analyzed the in vivo formation of atherosclerosis. Song et al. [9] showed that circANRIL overexpression was associated with the formation of atherosclerotic plaques and thrombi in rats that were fed a high-fat diet and were injected with a large dose of vitamin D3 (to promote arterial calcification) [9]. However, as extensively demonstrated in this review, circRNA molecule expression can correlate with either promotion or attenuation of atherosclerosis and therefore does not establish a causative relationship. Min et al. [123] showed increased expression of ciPVT1 in the senescent umbilical vein and coronary artery ECs, while silencing ciPVT1 led to delayed senescence, promoted proliferation, and increased the angiogenic activity of ECs. A correlative in vivo mouse study using a plug assay found that plugs mixed with silenced ciPVT1-transfected HUVECs showed less new vessel formation macroscopically [123]. This study shows the potential of the findings of in vivo studies to corroborate those of in vitro analyses of circRNA interactions and their effects on atherosclerosis [123]. However, such a model was rarely used in these investigations.

### 4.2. Off-Target RNA Silencing

This review identified a significant overlap of circRNA and miRNA interactions, resulting in disparate and opposing effects on mechanisms associated with the development of atherosclerosis. As seen in Figure 2, Figure 3 and Figure 4, the most investigated circRNA molecules, circ_USP36/circ_0003204, circCHFR, and circHIPK3, were found to have disparate, opposing, and often contradictory results across studies. While the majority of miRNAs inhibited by circ_USP36/circ_0003204 led to the regulation of genes that promoted pathogenic mechanisms such as increased proliferation, migration of cells, and inflammation, the sponging of others was found to be correlated with the opposite effects (Figure 2). Similar findings of harmful, protective, and equivocal effects on atherosclerosis development were seen when analyzing the mechanisms of circCHFR (Figure 3) and circHIPK3 (Figure 4) across studies.

Sponging the same miRNA by different circRNAs also had contradictory effects on cell proliferation, apoptosis, and inflammation. For example, miR-182-5p was demonstrated to be affected downstream of four different circRNA molecules: circ_USP36, circMTO1, hsa_circ_0004831, and Circ_0050486 [42,57,77,142]. While sponging of miR-182-5p by circ_USP36 led to increased activity of the KLF5 gene, which induced VSMC proliferation and metastasis, inhibition of miR-182-5p via overexpression of circMTO1 and subsequent RASA1 gene activation had the opposite effects of decreased VSMC proliferation and decreased apoptosis (Table S1). As another example, silencing of overexpressed circCHFR molecules led to de-inhibition of miR-370, allowing it to prevent expression of FOXO1/cyclin D1 genes, resulting in decreased proliferation and migration of VSMCs [21]. Circ-BANP silencing, which similarly resulted in increased levels of miR-370, however, was ultimately associated with the opposite finding: increased EC migration, invasion, and tube formation [45]. Similar results were seen when looking at the different effects of circHIPK3 and circ_0002194 on the sponging of mir-637 (Figure 4). All these cases underline the ubiquitous collateral off-target downstream and lateral effects of targeting a particular circRNA or miRNA for therapeutic purposes.

### 4.3. Differential Effects across Cell Types and Diseases

There were also significant cell-specific effects observed on the process of atherosclerosis development. Ox-LDL-treated HUVECs were associated with reduced expression of circHIPK3 in vitro, while overexpression correlated with reduced lipid accumulation and the promotion of autophagy [37]. In contrast, increased proliferation and reduced apoptosis of VSMCs, most likely a pathogenic mechanism, were observed in conjunction with increased circHIPK3 expression of aortic and umbilical artery VSMCs in vitro [61]. In response to a high-glucose environment, mouse aortic EC-secreted exosomes also promoted proliferation and inhibited apoptosis of VSMCs while promoting VCAM-1 expression and uptake of exosomes by VSMCs [82]. In human VSMCs, circHIPK3 was downregulated in tissues and blood samples of atherosclerosis patients and VSMCs with osteogenic and cartilage differentiation. Concordantly, overexpression of circHIPK3 was associated with the athero-protective effects of inhibited osteogenic and chondrogenic differentiation and reduced cell mineralization and calcium content [143].

Thus, increased expression of circHIPK3 was associated with both protective and detrimental mechanisms in the context of atherosclerosis development. The effects likely depend on particular cell types tested, e.g., VSMCs, ECs, and/or atherosclerosis-inducing agents, and overall milieus. Opposing effects in different cells further complicate the selection of cricRNA molecules such as circHIPK3. In this particular case, these studies suggest that silencing of circHPIK3 would lead to the negative effects of increased lipid accumulation in ECs and calcification of VSMCs but the positive effects of reduced proliferation and increased apoptosis in VSMCs, as well as decreased VCAM-1 expression and VSMC adhesion, indicating contrasting effects across different cell types.

Furthermore, it is likely that targeting a specific disease process, such as atherosclerosis in this case, may have unintended effects on other cardiovascular pathologies. While sponging of miR-370 by circCHFR led to increased FOXO1/Cyclin D activity which enhanced VSMC proliferation and migration, inhibition of miR-370 was also associated with beneficial effects on sinus node function in an in vitro mouse model of heart failure [21,151]. Thus, therapy aimed at silencing circCHFR to mitigate atherosclerosis development would likely lead to increased miR-370 expression, which may have pathogenic effects on sinus rhythm function in patients with heart failure. In addition, there are numerous extra-cardiac disease processes that may be affected by such genetic manipulation, the effects of which are hard to account for. For example, miR-370 has also been shown to play a regulatory role in various cancers, including cervical, ovarian, lung, gastric, and hepatocellular, among many others [156,157,158,159,160].

In summary, silencing of a particular circRNA leading to disinhibition of its related miRNA could result in the intended effect of halting atherosclerosis. However, several other pathways would need to be accounted for to mitigate the unintended consequences of amplifying atherosclerosis or other disease progressions. These include disparate, opposing, and contradictory downstream and lateral effects of silencing a particular circRNA in different tissue types and varying disease processes. Any risk-benefit analysis aimed at evaluating the adoption of such a therapeutic approach would ultimately be limited by the sheer scope of genetic interactions and their effects, as well as the discovery and knowledge of those mechanisms and effects.

## 5. Conclusions

This review represents the largest and most systematic review of studies evaluating the role of circRNA in the pathogenesis of atherosclerosis. With a focus on the most studied molecules, many disparate, opposing, and contradictory effects were observed across experiments. These include levels of the expression of a particular circRNA in atherosclerotic environments, attempted ascertainment of the in toto effects of circRNA or miRNA silencing on atherosclerosis progression, and off-target, cell-specific, and disease-specific effects. Accordingly, many of these studies conclude that a specific circular RNA regulates atherosclerosis. This review shows that this regulation is a complex orchestration more akin to directing traffic with multiple moving vehicles and intersections than a linear assembly line. Given the high potential for detrimental and unpredictable off-target effects downstream of circRNA manipulation, the practice of therapeutic targeting of circRNA or miRNA molecules appears too complex at the current level of knowledge. Future studies need to pay attention to the mechanisms being examined and manipulated in the context of stages of atherosclerosis, cell type, and downstream and lateral effects of circRNA manipulation. In this regard, we need more correlative in vivo studies designed to investigate the role of circRNAs in atherosclerosis development and progression.

## Data Availability

No new data were created or analyzed in this study. Data sharing is not applicable to this article.

## Supplementary Materials

The following supporting information can be downloaded at: https://www.mdpi.com/article/10.3390/jcm12134446/s1, Table S1: A summary of the mechanisms of the 140 studies investigating the role of circular RNA in the pathogenesis of atherosclerosis from the years 2016 to 2022, as identified in PubMed.
