# Supplementary material for: Circular RNA as Therapeutic Targets in Atherosclerosis: Are We Running in Circles?
Source: J Clin Med. 2023 Jul 2;12(13):4446. doi: 10.3390/jcm12134446 (PMC10342353; doi:10.3390/jcm12134446)
Supplement: Supplementary file 1 [file jcm-12-04446-s001.zip › jcm-2435350-supplementary.pdf]

Table S1. A summary of the mechanisms of the 140 studies investigating the role of circular RNA in the pathogenesis of atherolscerosis from the years 2016-2022 as identified in PubMed

Circular RNA	Cell type	Model	Disease/ Intervention	Expression alteration	Associated microRNA	Targeted Gene/Protein	Effect of overexpression
circANRIL [Holdt 2016]	EC, SMC, and adventitial fibroblasts	Human, in vitro	N/A	↑	independent of CDKN2A/B and miRNA sponging	Directly binds to PES1	impaired ribosome biogenesis, leading to activation of p53 and a subsequent decrease in proliferation, and increase in apoptosis
circWDR77 [Chen J 2017]	Vascular SMCs,	Human, in vitro	High-glucose	↑	miR-124	FGF-2	Increased proliferation and migration
hsa_circ_0003575 [Li CY 2017]	Vascular ECs	Human, in vitro	Ox-LDL	↑	miR-199-3p, miR-9-5p, miR377-3p or miR-141-3p	-	decreased proliferation and angiogenesis
circANRIL [Song CL 2017]	Vascular ECs	Rat, in vivo	high-fat diet/High-dose Vitamin D3	↑	N/A	-	increased vascular EC apoptosis and inflammatory factor expression
circACTA2 [Sun Y 2017]	Aortic SMCs	Humans, Mouse, Rat In vitro	TGF-β	↑	miR-548f-5p	NRG-1-ICD, α-SMA,	Increased stress fiber formation and cell contraction Leading to intimal hyperplasia
circ-SATB2 [Mao YY 2018]	Vascular SMCs	Human, in vitro	PDGF-BB	↑	miR-939	STIM1	Increased cell migration and proliferation, decreased apoptosis
Circ_0054633 [Pan 2018]	Umbilical vein ECs	Human, in vitro	High glucose	↑	miR-218	ROBO1, HO-1	Promoted proliferation, migration, tube formation, and inhibited apoptosis
CircHECW2 [Yang 2018]	Brain microvascular ECs	Human, in vitro, mouse in vivo	Methamphetamine or LPS	↑	miR-30D	ATG5	Promoted endothelial-mesenchymal transition
hsa_circ_0068087 [Cheng 2019]	Umbilical vein ECs	Human, in vitro	High-glucose	↑	miR-197	TLR4	Increased EC dysfunction, promoted inflammation, including NF-κB
circ_Lrp6 [Hall IF 2019]	Popliteal artery / Mouse carotid artery SMCs	Human in vitro, Mice in vivo	PDGF-BB	↑	miRNA-145	-	circ_Lrp6 buffers miR-145 activity when it is above a certain level of expression leading to decreased intimal hyperplasia
Circ-Sirt1 [Kong 2019]	Umbilical vein ECs / Vascular SMCs	Human/mouse in vivo	PDGF-BB or TNF-α	↓	miR-132 /212	Sirt1	Reduced VCAM-1, ICAM-1 and MCP-1 expression, inhibited inflammatory phenotypic switching of VSMCs

Table S1. A summary of the mechanisms of the 140 studies investigating the role of circular RNA in the pathogenesis of atherolscerosis from the years 2016-2022 as identified in PubMed

circ_0003575 [Shang L 2019]	Aortic ECs	Rat in vitro	Apoe-/- mice fed HFD	↑	miR-148a-3p	FOXO4 and FOXO3 expression	Promoted EC proliferation and migration
circRNA-0044073 [Shen L 2019]	Vascular SMCs, ECs	Human, in vitro	LPS	↑	miR-107	JAK/STAT signaling pathway	Promoted migration and proliferation
circ_RUSC2 [Sun J 2019]	Vascular SMCs	Human, in vitro	PDGF-BB	↑	miR-661	SYK	Promoted proliferation and migration
circNRG-1 [Sun Y 2019]	Aortic SMCs	Mice, in vitro	Ang II	↓	miR-193b-5p	NRG-1	reversed the inhibitory effect of Ang II on apoptosis
circCHFR [Yang L 2019]	Vascular SMCs	Human, in vitro	Ox-LDL	↑	miR-370	FOXO1/Cyclin D1	Increased proliferation and migration
hsa_circ_0002579 [Chen W 2020]	Aortic SMCs	Human, in vitro	PDGF-BB	↑	-	HMGA2	Promoted proliferation
circ_0010283 [Ding P 2020]	Vascular SMCs	Human, in vitro	Ox-LDL	↑	miR-370-3p	HMGB1	Increased viability and migration
hsa-circ_0010283 [Feng Z 2020]	Vascular SMCs	Human, in vitro	Ox-LDL	↑	miR-133a-3p	PAPP-A	Increased proliferation, migration, and invasion
circ_0029589 [Guo M 2020]	Peripheral blood mononuclear cells	Human, in vitro	ACS	↑	-	IRF-1	Decreased macrophage pyroptosis
circ-RELL1 [Huang H-S 2020]	Umbilical vein ECs	Human, in vitro	Ox-LDL	↑	miR-6873-3p	MyD88	Increased ICAM1 and VCAM1 expression, and inflammatory markers, i.e. NF-kB
hsa_circ_0029589 [Huang Z 2020]	Vascular SMCs	Human in vitro	Ox-LDL	↑	miR-214-3p	STIM1	Increased proliferation, migration, and invasion
hsa_circ_0000345 [Liu H 2020]	Aortic SMCs	Human in vitro	Ox-LDL	↓	miR-640	HIF-1α	Improved cell viability, reduced apoptosis, cycle arrest, cell invasion, supported HIF-1α
hsa_circ_0003204/ USP36 [Liu H 2020]	Umbilical vein ECs	Human, in vitro	Ox-LDL	↑	-	E-cadherin, N-cadherin, vimentin	Reduced proliferation and migration, increased expression of E-cadherin but reduced that of N-cadherine and vimentin
circCDR1as [Ma C 2020]	pulmonary artery SMCs	Human, in vitro	Hypoxia, calcified pulmonary arteries	↑	miR-7-5p	CAMK2D, CNN3	Increased differentiation to osteogenic phenotype promoting calcification

Table S1. A summary of the mechanisms of the 140 studies investigating the role of circular RNA in the pathogenesis of atherolscerosis from the years 2016-2022 as identified in PubMed

circDHCR24 [Peng W 2020]	Aortic vascular SMCs	Human, in vitro	PDGF-BB	↑	miR-149-5p	MMP9	Facilitated proliferation, migration, and phenotypic switching
circ_0003645 [Qin M 2020]	Umbilical vein ECs	Human, in vitro	Ox-LDL	↑	-	NF-κB	Increased inflammation and apoptosis, decreased cellular viability
Circ-ADAM9 [Tian 2020]	Progenitor ECs, ischemic limb	Human in vitro, mice in vivo	High-glucose	↑	miR-20a-5p	PTEN/ATG7	Promoted autophagy and apoptosis
circ_0124644 [Wang G 2020]	Vascular ECs	Human, in vitro	Ox-LDL	↑	miR-149-5p	PAPP-A	Promoted endothelial injury
circ-0077930 [Wang S 2020]	Umbilical vein SMCs	Human, in vitro	High-glucose	↑	miR-622	Kras CeRNA network	Induced senescence
circVEGFC [Wei H 2020]	Umbilical vein ECs	Human, in vitro	High-glucose	↑	miR-338-3p	HIF-1α	Promoted proliferation and apoptosis
circHIPK3 [Wei MY 2020]	Umbilical vein ECs	Human, in vitro, mouse in vivo	Ox-LDL	↓	miR-190b	ATG7 signal pathway	Reduced lipid accumulation and promoted autophagy
circRSF1 [Wei Z 2020]	Aortic ECs	Human, in vitro	Ox-LDL	↓	miR-758	CCND2	Increased cell viability, tube formation and migration, attenuated apoptotis
circ_0029589 [Yu H 2020]	Vascular SMCs	Human, in vitro	Ox-LDL	↑	miR-424-5p	IGF2	Induced proliferation, migration, invasion; reduced apoptosis
circPTPRA [Zhang LL 2020]	Vascular SMCs	Human, in vitro	Ox-LDL	↑	miR-636	SP1	promoted proliferation and repressed apoptosis
circBPTF [Zhang W 2020]	Umbilical vein ECs	Human, in vitro	High-glucose	↑	miR-384	LIN28B	Decreased cell viability, increased apoptosis, inflammation, oxidative stress
circ_USP36 [Zhao Q 2020]	Umbilical vein SMCs	Human, in vitro	Ox-LDL	↑	miR-182-5p	KLF5	Induced proliferation and metastasis
circ_CHFR [Zhuang J-B 2020]	Vascular SMCs	Human, in vitro	Ox-LDL	↑	miR-214-3p	Wnt3	Promoted cell growth, migration, and inflammation
circLRP6 [Bai Y 2021]	Vascular SMCs	Human, in vitro	High-glucose	↑	miR-545-3p	HMGA1	Promoted proliferation, migration and invasion
Circ-BANP [Chen G 2021]	Umbilical vein ECs	Human, in vitro	Ox-LDL	↑	miR-370	TXNIP	Reduced proliferation, migration, invasion, tube formation; increased inflammation and apoptosis

Table S1. A summary of the mechanisms of the 140 studies investigating the role of circular RNA in the pathogenesis of atherolscerosis from the years 2016-2022 as identified in PubMed

circ_UBR4 [Ding Y 2021]	Vascular SMCs	Human, in vitro	Ox-LDL	↑	MiR-637	FOXO4	Promoted cell cycle progression, proliferation, and migration
circ_0004872 [Fan 2021]	Aortic vascular SMCs	Human, in vitro	PDGF-BB	↑	MiR-513a-5p	TXNIP	Induced proliferation, migration and dedifferentiation
circMAPK1 [Fu 2021]	Vascular SMCs	APOE-/- mice in vitro	High-fat diet	↑	miR-22-3p	MECP2	Increased proliferation and migration
circ_0093887 [Gao 2021]	ECs	Human, in vitro	Ox-LDL	↓	miR-876-3p	CCND2 SUCNR1	abrogated cell injury, decreased cell proliferation and migration
circ_0090231 [Ge 2021]	Aortic ECs	Human, in vitro	Ox-LDL	↑	miR-635	NLRP3	improved cell viability, reduced cell injury and pyroptosis
circEsyt2 [Gong 2021]	Vascular SMCs	APOE-/- mice, in vivo	High-fat diet	↑	-	Directly interacted with PCBP1	enhanced cell proliferation and migration and inhibited apoptosis and differentiation
hsa_circ_0004543 [Han 2021]	umbilical vein ECs	Human, in vitro	Ox-LDL	↑	-	PI3K/AKT/NOS3	Reduced EC proliferation, migration, and invasion; promoted apoptosis
circSCAP [He 2021]	Vascular SMCs	Samples of patients with AS	Ox-LDL	↑	miR-221-5	PDE3B	Facilitated lipid deposition, inflammation, and oxidative stress
circDIP2C [Hu F 2021]	Umbilical vein ECs	Human, in vitro	Ox-LDL	↓	microRNA-556-5p	TET2	Promoted cell viability and angiogenesis, reduced oxidative stress and cytotoxicity
circUSP36 [Huang JG 2021]	Aortic ECs	Human, in vitro	Ox-LDL	↑	miR-637	WNT4	suppressed proliferation and migration of ECs
circ_0074673 [Huang Y 2021]	Umbilical vein ECs	Human, in vitro	Gestational Diabetes	↑	miR-1200	MEOX2	suppressed proliferation, migration, and angiogenesis
circMTO1 [Ji N 2021]	Vascular SMCs	Human, in vitro	Ox-LDL	↓	miR-182-5p	RASA1	inhibited proliferation, promoted apoptosis
circ_0004104 [Ji P 2021]	Vascular ECs	Human, in vitro	Ox-LDL	↑	miR-100	TNFAIP8	inhibited proliferation, promoted apoptosis and inflammation
circUSP9X [Jiang X 2021]	Umbilical vein ECs	Human, in vitro	Ox-LDL	↑	miR-148b-3p	KLF5	Promoted LDH leakage, apoptosis, inflammation, and oxidative stress
circ-UBAP2 [Jiewei 2021]	Retinal microvascular ECs	Human, in vitro	High-glucose	↑	miR-589-5p	EGR1	Induced oxidative stress and dysfunction
circHIPK3 [Kang 2021]	Aortic/ umbilical artery SMCs	Human, in vitro	-	↑	miR-637	CDK6	Enhanced SMC proliferation, reduced apoptosis, promoted cell cycle progression

Table S1. A summary of the mechanisms of the 140 studies investigating the role of circular RNA in the pathogenesis of atherolscerosis from the years 2016-2022 as identified in PubMed

circ_0065149 [Li D 2021]	Umbilical vein ECs	Human, in vitro	Ox-LDL	↓	miR-330-5p	NF-κBp65	Promoted cell viability, cell migration and invasion; inhibited inflammation and apoptosis
circ_0002984 [Li R 2021]	Vascular SMCs	Human, in vitro	Ox-LDL	↑	miR-326-3p	VAMP3	Increased VSMC viability, cell cycle distribution, and migration capacity
circ_0068087 [Li S 2021]	Umbilical vein ECs	Human, in vitro	Ox-LDL	↑	miR-186-5p	ROBO1	Decreased cellular viability, increased apoptosis and inflammation
circ_GRN [Li X 2021]	Vascular SMCs	Human, in vitro	Ox-LDL	↑	miR-214-3p	FOXO1	Promoted proliferation, migration, inflammation
circSMARCA5 [Liang 2021]	Umbilical vein ECs	Human, in vitro	Ox-LDL	↓	-	SRSF1	reversed oxLDL-induced inhibition of EC proliferation, promoted tube formation
circ_ROBO2 [Lin 2021]	Aortic SMCs	Human, in vitro	PDGF-BB	↑	miR-149	TRAF6/NF-κB	Promoted proliferation and migration, suppressed apoptosis, increased NF-κB signaling
circPPP1CC [Liu J 2021]	Umbilical vein ECs	Human, in vitro	Lipopolysaccharide	↑	miR-103a-3p, miR-107	HMGB1, TLR9, AIM2	Induced pyroptosis
circSFMBT2 [Luo Y 2021]	Vascular SMCs	Human, in vitro	PDGF-BB	↑	miR-331-3p	HDAC5	Induced proliferation, migration
circACTA2 [Ma 2021]	Vascular SMCs	Human, in vitro	Ang II	↑	CDK4 mRNA	CDK4, ILF3	Induced senescence
circSOD2 [Mei 2021]	Aortic vascular SMCs, carotid balloon injury	Human, in vitro, rat in vivo	PDGF-BB	↑	miR-206	NOTCH3	Promoted SMC proliferation and neointima formation
circUSP36 [Miao 2021]	Umbilical vein ECs	Human, in vitro	Ox-LDL	↑	miR-20a-5p	ROCK2	Induced cell cycle arrest, apoptosis, inflammatory responses, cell migration and invasion
circ-USP9X [Peng H 2021]	Umbilical vein ECs	Human, in vitro	Ox-LDL	↑	miR-599	CLIC4	Reduced viability, suppressed cell cycle progression and angiopoiesis, and increased apoptosis, inflammation, and oxidative stress
circ_USP36 [Peng K 2021]	Umbilical vein ECs	Human, in vitro	Ox-LDL	↑	miR-98-5p	VCAM1	Accelerated apoptosis, inflammation, reduced viability
hsa_circ_0003204 [Peng W 2021]	Carotid artery ECs	Human, in vitro	Ox-LDL	↑	miR-188-3p	TRPC6	Reduced viability; promoted apoptosis, inflammation and oxidative stress

Table S1. A summary of the mechanisms of the 140 studies investigating the role of circular RNA in the pathogenesis of atherolscerosis from the years 2016-2022 as identified in PubMed

circ_0000231 [Shao 2021]	Umbilical vein ECs	Human, in vitro	Ox-LDL	↑	MiR-135a-5p	CLIC4	Antagonized effects of astragaloside to suppress apoptosis, inflammation, and oxidative stress, recover viability
hsa_circ_0004831 [Su G 2021]	Umbilical vein ECs	Human, in vitro	Ox-LDL	↑	miR-182-5p	CXCL12	Attenuated effects of atorvastatin to promote cell viability and cell cycle progression, inhibit apoptosis, oxidative stress and inflammation
circ_0003204 [Su Q 2021]	Umbilical vein ECs	Human, in vitro	Ox-LDL	↑	miR-330-5p	TLR4	suppress viability and promote apoptosis, inflammatory response, and oxidative stress
circUBR4 [Sun C 2021]	Vascular SMCs	Human, in vitro	Ox-LDL	↑	miR-185-5p	FRS2	Promoted proliferation and migration
circ_0000345 [Tiliwaldi 2021]	Umbilical vein ECs	Human, in vitro	Ox-LDL	↓	miR-129-5p	TET2	promoted cell viability and cell cycle progression and hampered cell apoptosis
circ_0003204 [Wan 2021]	Umbilical vein ECs	Human, in vitro	Ox-LDL	↑	miR-942-5p	HDAC9	Reduced cell viability, increased apoptosis, oxidative stress and inflammation
circHIPK3 [Wang S 2021]	Aortic EC-secreted exosomes	Mouse, in vitro	High-glucose	↑	miR-106a-5p	FOXO1/VCAM-1	Promoted proliferation and inhibited apoptosis of VSMCs, promoted VCAM-1 expression and uptake of exosomes by VSMCs
circTM7SF3 [Wang X 2021]	THP-1-derived macrophages	Human, in vitro	Ox-LDL	↑	miR-206	ASPH mRNA	Suppressed viability, promoted apoptosis, inflammation and oxidative stress of THP-1-derived macrophages
circRNA-006896 [Wen 2021]	Umbilical vein ECs	Human, in vitro	unstable atherosclerotic plaques	↑	miR-1264	DNMT1	Amplified proliferation and migration of ECs in serum of patients with unstable as compared to stable plaque
circ_CHFR [Wu 2021]	Brain microvascular ECs	Human, in vitro	Ox-LDL	↑	miR-15a-5p	EGFR	Increased cell proliferation, cell cycle, apoptosis
circGNAQ [Wu WP 2021]	ECs	Human, in vitro	Senescence	↓	miR-146a-5p	PLK2	Suppressed EC senescence, reduced cell proliferation and angiogenesis
circNPHP4 [Xiong 2021 F]	monocyte-derived sEVs from CAD patients	Human, in vitro	CAD	↑	miR-1231	EGFR	Facilitated adhesion of monocytes and coronary artery ECs, increased expression of ICAM-1 and VCAM-1

Table S1. A summary of the mechanisms of the 140 studies investigating the role of circular RNA in the pathogenesis of atherolscerosis from the years 2016-2022 as identified in PubMed

circDENND1B [Xu F 2021]	aorta	ApoE −/− mice, in vivo	High-fat diet	↓	miR-17-5p	Abca1	Promoted cholesterol efflux from macrophages alleviating foam cell formation
circ_0003423 [Yu H 2021]	Brain microvascular ECs	Human, in vitro	Ox-LDL	↓	miR-589-5p	TET2	Promoted cell viability and tube formation, reduced apoptosis, oxidative injury
hsa_circ_0030042 [Yu F 2021]	Umbilical vein ECs	Human, in vitro	Ox-LDL	↓	Sponged eIF4A3	FOXO1/beclin1	inhibited ox-LDL-induced abnormal autophagy and promoted plaque stability
circMAP3K5 [Zeng Z 2021]	Coronary artery SMCs, wire-injury femoral arteries	Human, in vitro, mouse in vivo	PDGF-BB	↓	miR-22-3p	TET2	inhibited SMC proliferation, reduced neointima formation
hsa_circ_0003204 [Zhang B 2021]	Primary aortic ECs	Human, in vitro	Ox-LDL	↑	miR-330-5p	Nod2	Facilitated oxidative stress and apoptosis
circ_0004104 [Zhang C 2021]	Umbilical vein ECs	Human, in vitro	Ox-LDL	↑	miR-328-3p	TRIM14	suppressed cell viability and angiogenic ability, promoted apoptosis, inflammation, and oxidative stress
circ_0010283 [Zhang P 2021]	Vascular SMCs	Human, in vitro	Ox-LDL	↑	miR-377-3p	CCND1	Increased proliferation, migration, and inflammation; inhibited apoptosis
Circ_CLASP2 [Zhang Q 2021]	Umbilical vein ECs	Human, in vitro	High glucose	↓	miR-140-5p	FBXW7	Promoted proliferation and inhibited apoptosis
circRSF1 [Zhang Xiaohao 2021]	Umbilical vein ECs	Human, in vitro	Ox-LDL	↓	miR-135b-5p	HDAC1	induced cell proliferation while inhibited apoptosis and inflammation
hsa_circ_0024093 [Zhang Xue 2021]	Vascular SMCs	Human, in vitro	PDGF-BB	↑	miR-889-3p	USP9X/YAP1	Promoted cell proliferation and migration, inhibited apoptosis
circ_USP36 [Zhang Y 2021]	Umbilical vein ECs	Human, in vitro	Ox-LDL	↑	miR-197-3p	ROBO1	Inhibited proliferation and cell cycle progression, triggered apoptosis and inflammation
circ_UBR4 [Zhang Y 2021]	Vascular SMCs	Human, in vitro	Ox-LDL	↑	miR-107	ROCK1	Promoted cell cycle, increased cell viability, colony-forming ability, and migration; increased expression of proliferating cell nuclear antigen and matrix metalloproteinase 2
circ_0002984 [Zheng 2021]	Vascular SMCs	Human, in vitro	Ox-LDL	↑	miR-379-5p	FRS2	induced proliferation, migration, and invasion

Table S1. A summary of the mechanisms of the 140 studies investigating the role of circular RNA in the pathogenesis of atherolscerosis from the years 2016-2022 as identified in PubMed

hsa_circ_0001445 [Cai 2022]	Umbilical vein ECs	Human, in vitro	Ox-LDL	↓	miRNA-640	TNF-α, IL-1β and IL-16	promoted cell proliferation, inhibited ox-LDL-induced inflammation, apoptosis
circ_TNPO1 [Chen M 2022]	Vascular SMCs	Human, in vitro	Ox-LDL	↑	miR-181b	Notch1	Promoted proliferation and migration
circ_0000345 [Chen S 2022]	Arterial SMCs	Human, in vitro	Ox-LDL	↓	Mir-647	PAPD5	Reduced cellular proliferation, migration and invasion, and inflammation
hsa_circ_0005699 [Chen T 2022]	Umbilical vein ECs	Human, in vitro/ ApoE-/- mice in vivo	Ox-LDL	↑	miR-450b-5p	NFKB1	Increased apoptosis and inflammation
hsa_circRNA_102541 (circ_102541) [Du N 2022]	Umbilical vein ECs	Human, in vitro	Athero-sclerosis	↑	miR-296-5p	PLK1	Augmented proliferation, reduced apoptosis
cZNF292/ cZfp292 [Heumüller 2022]	Umbilical vein ECs	Human, in vitro/ mice in vivo	Laminar flow / cZfp292−/− mice	↑	-	SDOS protein	Promoted laminar flow-induced alterations in cell orientation
hsa_circ_0008896 [Hou 2022]	Vascular SMCs	Human, in vitro/ mice in vivo	Ox-LDL / femoral artery wire injury	↑	hsa-miR-633	CDC20B	Increased proliferation, migration, and invasion of vascular SMCs
circ_0033596 [Jing 2022]	Umbilical vein ECs	Human, in vitro	Ox-LDL	↑	miR-217-5p	CLIC4	Inhibited viability and cell cycle progression, promoted apoptosis
circTEX14 [Kou 2022]	Vascular SMCs	Human, in vitro	Ox-LDL	↓	miR-6509-3p	THAP1	Inhibited proliferation and migration and enhanced apoptosis
circ_0090231 [Lei 2022]	Umbilical vein ECs	Human, in vitro	Ox-LDL	↑	miR-9-5p	TXNIP	Impaired angiogenesis, reduced inflammation and oxidative stress, increased apoptosis
circCHMP5 [Li X 2022]	Umbilical vein ECs	Human, in vitro	Ox-LDL	↑	miR-532-5p	ROCK2	suppressed cell cycle, proliferation, and angiogenesis, induced apoptosis and inflammation
circCHFR [Li Y 2022]	Umbilical vein ECs	Human, in vitro	Ox-LDL	↑	miR-15b-5p	GADD45G	Promoted apoptosis and proinflammatory cytokine secretion, reduced cell survival
hsa_circ_0021155 [Lin J 2022]	Aortic SMCs	Human, in vitro	Ox-LDL, high-glucose	↑	miR4459	TRPM7	promoted the proliferation and migration of cells

Table S1. A summary of the mechanisms of the 140 studies investigating the role of circular RNA in the pathogenesis of atherolscerosis from the years 2016-2022 as identified in PubMed

circIRAK1 [Liu F 2022]	Umbilical vein ECs	Human, in vitro	Ox-LDL	↑	miR-330-5p	TRIM14	Reduced cell viability and angiogenesis, enhanced apoptosis, inflammation, and oxidative stress
circ_0026218 [Liu J]	Umbilical vein ECs	Human, in vitro	Ox-LDL	↑	miR-188-3p	TLR4/NF-κB	Suppressed cell proliferation, increased cell apoptosis, inflammation, and oxidative stress
hsa_circ_0022742 [Liu S 2022]	Umbilical vein ECs	Human, in vitro	High-glucose	↓	miR-503-5p	FBXW7	suppressed apoptosis, abnormal vascular differentiation, and secretion of inflammatory factors
circCHFR [Lu X 2022]	Aortic vascular SMCs	Human, in vitro	Ox-LDL	↑	miR-214-3p	PAPP-A	Induced proliferation, migration, and invasion
circPTPRA [Luo 2022]	Umbilical vein ECs	Human, in vitro	Ox-LDL	↑	miR-671-5p	-	Reduced in cell viability, increase in apoptosis, and enhanced inflammation
hsa_circ_0030042 [Ma J 2022]	Vascular SMCs	Human, in vitro	TNF-α	↑	miR-514a-3p	FOXO1	Promoted proliferation and migration, reduced apoptosis
circPCNX [Ma W 2022]	Aortic vascular SMCs	Human, in vitro	PDGF-BB	↑	miR-1278	DNMTI	Induced cell proliferation, cell cycle progression, and migration
circ_0124644 [Mao 2022]	Umbilical vein ECs	Human, in vitro	Ox-LDL	↑	miR-370-3p	FOXO4	Inhibited cell viability, proliferation and angiogenesis, promoted apoptosis and inflammation
circ_0002194 [Mei R 2022]	Vascular ECs	Human, in vitro	Ox-LDL	↑	miR-637	PACS2	Reduced cell viability and angiogenesis, promoted cell apoptosis and oxidative stress
ciPVT1 [Min 2022]	Umbilical vein /coronary artery ECs, plug assay	Human, in vitro, mice in vivo	Senescence	↓	miR-24-3p	CDK4	Delayed senescence, promoted proliferation, and increased angiogenic activity
circUBR4 [Peng 2022]	Vascular SMCs	Human, in vitro	Ox-LDL	↑	miR-491-5p	NRP2	Promoted proliferation and migration
circGSE1 [Qiu 2022]	Old ECs, ischemic limbs	Human, in vitro / mice in vivo	Senescence	↓	miR-323-5p	NRP1	Promoted proliferation, migration, tube formation
circSmoc1-2 [Ryu 2022]	Vascular SMCs	Human, in vitro / mice in vivo	Vascular calcification	↓	miR-874-3p	Adam19	Reduced vascular calcification

Table S1. A summary of the mechanisms of the 140 studies investigating the role of circular RNA in the pathogenesis of atherolscerosis from the years 2016-2022 as identified in PubMed

circRNA-0024103 [Tian 2022]	Umbilical vein ECs	Human, in vitro	Ox-LDL	↑	miR-363	MMP-10	Promoted proliferation, migration, invasion, and tube-formation, inhibited apoptosis
circ_CHFR [Wang M 2022]	Vascular SMCs	Human, in vitro	PDGF-BB	↑	miR-149-5p	NRP2	Promoted cell proliferation, migration, invasion, and cell apoptosis
circ_0003423 [Wang P 2022]	Umbilical vein ECs	Human, in vitro	Ox-LDL	↓	miR-142-3p	SIRT3/SOD2	Promoted proliferation, migration, and angiogenesis, reduced oxidative stress and apoptosis
circ_0002984 [Wang X 2022]	Vascular SMCs	Human, in vitro	Ox-LDL	↑	miR-181b-5p	VEGFA	Induced proliferation and migration
circ_0093887 [Wang Y 2022]	Aortic ECs	Human, in vitro	Ox-LDL	↓	miR-758-3p	BAMBI	promoted cellular viability and tube formation, restrained cell apoptosis
circ_ARHGAP32 [Wang Y 2022]	Vascular SMCs	Human, in vitro	Ox-LDL	↑	miR-665	FGF-2	Induced proliferation and migration
hsa_circ_0000280 [Wang Z 2022]	Aortic SMCs	Human in vitro, murine ex vivo	PDGF-BB	↓	CDKN1A mRNA	ELAVL1	Reduced proliferation and cell cycle progression
mmu_circ_0000271 [Yang J 2022]	Vascular SMCs	Mouse, in vitro	Ox-LDL	↑	miR-5123	-	promoted viability, angiogenesis and invasion, proliferation, and migration
circLMF1 [Yang Y 2022]	Aortic vascular SMCs	Human, in vitro	PDGF-BB	↑	miR-125a-3p	VEGFA/FGF1	Promoted cell viability, cell cycle progression, and migration
Has_circ_0007478 [Ye B 2022]	Macrophages	Human, in vitro	Ox-LDL	↑	miR-765	EFNA3	Increased inflammation and macrophage uptake of ox-LDL
circ_ROBO2 [Ye Q 2022]	Cardiac microvascular ECs	Human, in vitro	Ox-LDL	↑	miR-186-5p	TRIM14	Suppressed cell proliferation and angiogenic ability, promoted apoptosis
circ_0030042 [Yu L 2022]	Umbilical vein ECs	Human, in vitro	Ox-LDL	↓	miR-616-3p	RFX7	Facilitated cell proliferation, repressed apoptosis, reduced inflammatory factors
circ_0003204 [Zhang D 2022]	Umbilical vein ECs	Human, in vitro	Ox-LDL	↑	miR-491-5p	ICAM1	Increased inflammation and cell apoptosis, reduced cell viability and tube formation ability
circLIFR [Zhang H 2022]	Umbilical artery SMCs	Human, in vitro	Ox-LDL	↓	miR-1299	KDR	Enhanced vascular proliferation, migration, invasion; impeded apoptosis

Table S1. A summary of the mechanisms of the 140 studies investigating the role of circular RNA in the pathogenesis of atherolscerosis from the years 2016-2022 as identified in PubMed

circ_0086296 [Zhang M 2022]	Carotid artery plaques, Umbilical vein ECs, aorta	Human, in vitro, Mouse in vivo	Ox-LDL	↑	miR-576-3p	IFIT1-STAT1	Promoted EC proliferation, migration, and inflammatory marker release
Circ_0050486 [Zhang P 2022]	Aortic ECs	Human, in vitro	Ox-LDL	↑	miR-182-5p	MYD88	Promoted endovascular injury
CircHIPK3 [Zhang W-B 2022]]	Vascular SMCs	Human, in vitro	AS	↓	miR-106a-5p	MFN2	inhibited osteogenic/ chondrogenic differentiation, reduced cell mineralization and calcium content
Circ_0004104 [Zhang Y 2022]	Umbilical vein ECs	Human, in vitro	Ox-LDL	↑	miR-942-5p	ROCK2	Reduced cell proliferation, augmented apoptosis
circITGB1 [Zhu J 2022]	Dendritic cells	Human, in vitro, mouse in vivo	ACS	↑	miR-342-3p	NFAM1	Regulated dendritic cell maturation and promoted cardiac damage

SMCs: smooth muscle cells, ox-LDL: oxidized low-density lipoprotein, Bcl-2: B-cell lymphoma 2, PES1: pescadillo homologue 1, FGF-2: fibroblast growth factor 2, NRG: neuregulin-1, ICD: intracellular domain, α -SMA: smooth muscle α-actin, DNMT3A: DNA (cytosine-5)-methyltransferase 3A, STAT3: Signal transducer and activator of transcription 3, STIM1: stromal interaction molecule 1, LPS: lipopolysaccharide, NRP1: neuropilin-1, LPS: lipopolysaccharide, AS: atherosclerosis, Ang II: Angiotensin II, VEGFR-2: vascular endothelial growth factor receptor-2, IGFR1: insulin-like growth factor 1 receptor, HMGA2: High-mobility group AT-hook 2, HMGB1: high mobility group box 1 protein, PAPP-A: pregnancy-associated plasma protein, ACS: acute coronary syndrome, IRF-1: IFN regulatory factor-1, MyD88: myeloid differentiation primary response 88 protein, CAMK2D: calcium/calmodulin-dependent kinase II-delta, CNN3: calponin 3, MMP9: matrix metallopeptidase 9. ceRNA – competing endogenous RNA, HIF-1α: hypoxia inducible factor 1 alpha, HIPK3: homeodomain interacting protein kinase 3, IGF2: insulin-like growth factor 2, LYPD3: LY6/PLAUR domain containing 3, GRIA4: glutamate ionotropic receptor, LIN28B: lin-28 homolog B, CKAP4: cytoskeleton-associated protein 4, KLF5: kruppel-like factor 5, TXNIP: thioredoxin-interacting protein, MCL1: myeloid cell leukemia sequence 1, PDGF-BB: platelet-derived growth factor-BB, CCND2: cyclin D2, SUCNR1: succinate receptor 1, NLRP3: NOD-, LRR- and pyrin domain-containing protein 3, PCBP1: polyC-binding, protein, PDE3B: phosphodiesterase 3B, DIP2C: disco interacting protein 2 homolog C, MEOX2: mesenchyme homeobox 2, RASA1: RAS P21 protein activator 1, TNFAIP8: TNF alpha induced protein 8, USP9X: ubiquitin-specific peptidase 9 X-linked, PDCD6: programmed cell death 6, EGR1: early growth response 1, VAMP3: vesicle-associated membrane protein 3, ROBO1: roundabout guidance receptor 1, SRSF1: serine/arginine-rich splicing factor 1, NF-kB – nuclear factor kappa-B, FNDC3B: fibronectin type III domain containing 3B, ADAM10: ADAM Metallopeptidase domain 10, HCAC5: histone deacetylase 5, CDK4: cyclin dependent kinase 4, ILF3: interleukin enhancer binding factor 3, THAP1: thanatos-associated domain-containing apoptosis-associated protein 1, ROCK2: Rho-associated coiled-coil kinase 2, CLIC4: chloride intracellular channel 4, VCAM1: vascular cell adhesion molecule 1, TRPC6: transient receptor potential canonical channel 6, KDR: kinase insert domain receptor, CXCL12: C-X-C motif chemokine 12, TLR4: toll-like receptor 4, FRS2: fibroblast growth factor receptor substrate 2, Grm1: glutamate metabotropic receptor 1, FUS: FUS RNA binding protein, TET2: ten-eleven translocation-2, HDAC9: histone deacetylases 9, DNMT1: DNA Methyltransferase 1, PACS2: Phosphofurin Acidic Cluster Sorting Protein 2, EGFR: epidermal growth factor receptor, PLK2: polo like kinase 2, sEVs: small extracellular vesicles, CAD: coronary artery disease, Abca1: ATP binding cassette subfamily A member 1, SOD2: superoxide dismutase-2, eIF4A3: eukaryotic initiation factor 4A-III, Nod2: nucleotide-binding oligomerization domain 2, TRIM14: t ripartite Motif Containing 14, CCND1: cyclin D1, HDAC1: histone deacetylase 1, YAP1: yes1-associated transcriptional

Table S1. A summary of the mechanisms of the 140 studies investigating the role of circular RNA in the pathogenesis of atherolscerosis from the years 2016-2022 as identified in PubMed

regulator, FRS2: factor receptor substrate 2, Notch1: neurogenic locus notch homolog protein, SDOS: significantly enriched protein, NFKB1: nuclear factor kappa beta subunit 1, PLK1: polo-like kinase 1, CDC20B: cell division cycle 20B, GADD45G: growth arrest and DNA damage inducible gamma, FBXW7: F-box and WD repeat domain containing 7, RFXY: regulatory factor X 7, NRP2: Neuropilin-2, VEGFA: SIRT3: Sirtuin 3, Vascular endothelial growth factor A, BAMBI: BMP And Activin Membrane-Bound Inhibitor, ICAM1: intracellular adhesion molecule 1, KDR: kinase insert domain receptor, IFIT1-STAT1: interferon induced protein with tetratricopeptide repeats 1- signal transducer and activator of transcription 1, NFAM1: NFAT activating molecule 1
